# Behavior of unsaturated sandy loess under high-frequency unilateral cyclic loading

**DOI:** 10.1038/s41598-025-16105-2

**Published:** 2025-08-26

**Authors:** Zhenwei Jiang, Bingyan Shen, Jiaqing Gao, Dong Xie, Zhenxing Han

**Affiliations:** College of Geological Engineering and Surveying, Chang’an University, Xi’an, China

**Keywords:** Unsaturated sandy loess, Dynamic characteristic, High-frequency cycle, Cumulative strain, Environmental sciences, Engineering

## Abstract

Sandy loess, which is distinguished by its granular composition dominated by sand particles and minimal clay, is vulnerable to structural destabilization under cyclic loading. This report focuses on recurrent geotechnical failures in the hydrocarbon extraction zones of China’s Loess Plateau. The investigation employs cyclic triaxial testing to evaluate the moisture-dependent and frequency-governed mechanical behaviors of unsaturated sandy loess, particularly in the context of the Maliancheng Village landslide event. Beyond the plastic limit threshold, the moisture content has negligible effects on the stress–strain hysteresis, shear rigidity, and energy dissipation of the system. However, sub-plastic-limit conditions induce strain amplification by increasing damping ratios and decreasing shear resistance. Meanwhile, the strain magnitude and shear stiffness increase proportionally with loading frequency, whereas damping responses have an inverse frequency relationship. Notably, high-rate cyclic loads have less of an influence on early-phase mechanical signatures. Progressive strain accumulation under sustained high-frequency loading follows triphasic evolution, i.e., exponential escalation, transitional refinement, and metastable equilibrium, while stabilization cycles escalate nonlinearly as a function of the applied stress.

## Introduction

Loess is found in many regions globally, and it accounts for roughly 10% of the world’s land area. In China, it is mainly distributed in the Loess Plateau, comprising Shaanxi and Shanxi, which are important areas for oil and gas production. Loess is an important component of foundations, slopes, and other structures, and it undergoes significant changes in terms of mechanical properties and characteristics under natural conditions (e.g., rainfall, earthquakes) and under dynamic loads from vehicles, subway, and mechanical vibrations^1–5^. During the extraction of oil and gas resources, the long-term cyclic vibration of drilling rigs and the special structure of loess^6,7^ causes structural damage to unsaturated sandy loess and dynamic liquefaction damage to saturated loess. This leads to a higher probability of geological disasters, such as landslides, during oil and gas extraction compared to such risks in other oil fields. This has a significant impact on engineering projects and exacerbates the damage to the ecological environment of the Loess Plateau^8–13^. Additionally, secondary disasters, such as collapse and deformation must not be underestimated^14,15^. Long-term investigations have recently revealed that multiple landslides have occurred in the oil field well sites in northern Shaanxi, all of which occurred during the operation of the wells. These landslides have caused significant losses. Notably, the loess in the areas where such landslides have occurred is mainly sandy loess. Therefore, it is crucial to better understand the dynamic characteristics of sandy loess under cyclic vibration to support engineering construction in this region.

Pioneering research into the characteristics of sandy loess began in the 1960s. Early studies^16,17^ indicated that the particle sizes across the Loess Plateau are not uniform and particles generally become finer from northwest to southeast. Chang et al.^18^ investigated the impacts of different sand contents on the mechanical characteristics of loess mixtures and explored the aspects that affect the shear strength of unsaturated soils. Tian et al.^19^ investigated the factors influencing the shear strength of unsaturated soils.

Studies probing the dynamic characteristics of loess mainly involve dynamic triaxial tests^20,21^, leading to a range of results^22–24^. Karam et al.^25^ conducted cyclic triaxial tests with loess from northern France. Ng et al.^26^ measured the shear modulus and other parameters of mixed loess samples using triaxial tests to determine the properties that improved the stability of loess samples. Liu et al.^27^ monitored cumulative deformation and constructed a constitutive model for measuring the cumulative strain of loess. This work provided a theoretical basis for subsequent studies of cumulative loess deformation^28–30^. However, reports describing the dynamic characteristics of unsaturated sandy loess have primarily focused on typical loess and cohesive loess, whereas there are few studies on the dynamic characteristics of sandy loess from different regions or with different composition. For oilfield engineering projects in areas with complex geological conditions, it is crucial to understand the dynamic characteristics and influencing factors of unsaturated sand loess, e.g. regarding rainfall and drilling rig vibrations, in order to prevent landslide disasters in areas with unsaturated sand loess.

This article aims to fill this knowledge gap by taking the Malincheng Village landslide in Yan’an City as the engineering background case; this landslide was triggered by drilling rig vibrations during construction. The pristine loess samples near the back wall of the Malincheng Village landslide were sampled and subjected to triaxial tests after indoor consolidation to explore the dynamic characteristics of unsaturated sandy loess. The impacts of moisture content and vibrational frequency on the dynamic stress-strain relationship, dynamic shear modulus, and damping ratio were determined experimentally. On this basis, the law governing sandy loess development under high cumulative vibrational strain was explored. The results provide a valuable reference for exploring the dynamic characteristics of sandy loess under cyclic vibration and for promoting landslide disaster prevention and reduction in loess areas.

## Materials and methods

### Materials

**Fig. 1:**
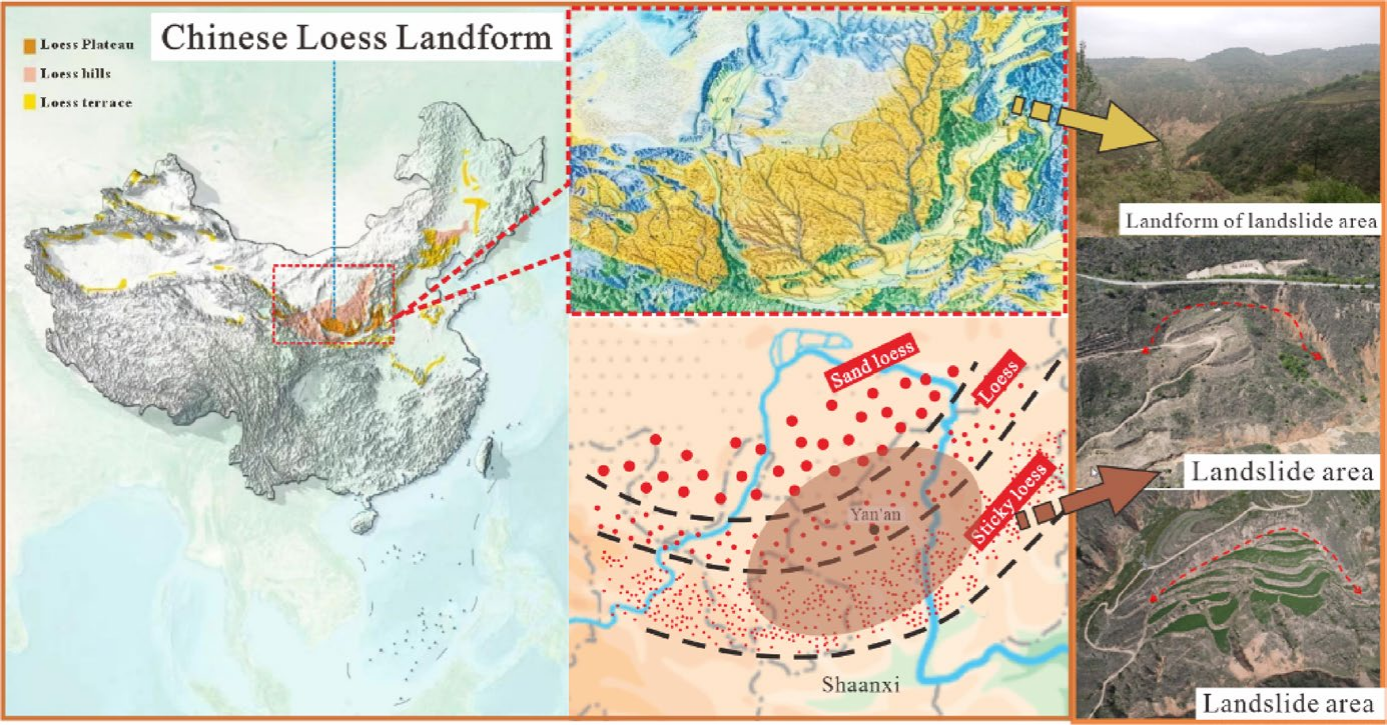
Landslide region including sampling area of sandy loess (The picture is from the “Atlas of Natural Geography of China” Liu Guangming, Beijing: China Map Publishing House, 2010) (The landslide image in the picture was taken by a DJI MAVIC AIR2 drone from DJI, which was used by the author Zhenxing Han).

**Fig. 2:**
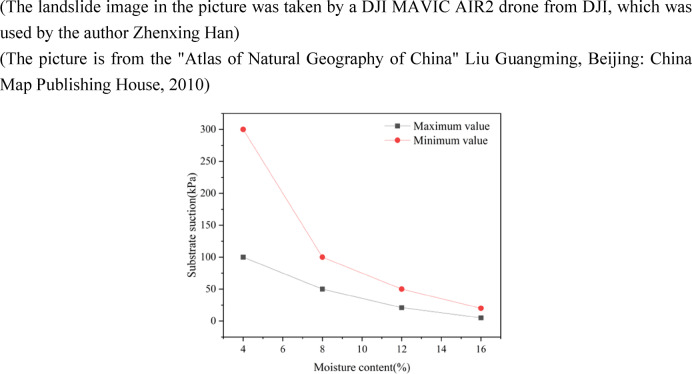
Soil–water retention curve.

The original loess near the back wall of the landslide in Maliancheng Village, Yan’an City, Shaanxi Province, China was used as the study object for the experiments. On site, undisturbed loess samples with a depth of 2–4 m were collected. The sampling area and sand loess zoning are shown in Fig. 1. After retrieving the sample, a cylindrical specimen with dimensions of 39.1 mm diameter and 80 mm height was created. The sample had a sand content of 37% and a clay content of 9.11%, indicating a high sand content characteristic of typical sandy loess. The physical parameters of the soil were determined mainly based on the “Standard for Soil Test Methods”, which specifies that during the preparation process, efforts should be made to avoid errors caused by disturbances to the sample. The sample reaches saturation when the moisture content reaches 14%, and the physical parameter indicators are shown in Table 1. The water retention curve of the soil is determined based on matric suction and moisture content. The soil water retention curve can be obtained based on the general characteristics of the unsaturated soil, the structural characteristics of the loess, and the moisture content of the soil, as shown in Fig. 2.

In this study, anaerobic water was injected into the unsaturated sand loess as the test fluid. The core characteristics of the test fluid are its low oxygen content and physical stability. In addition to reducing the oxygen content, anaerobic water usually contains no other impurities and is chemically stable. In addition, this test fluid has wide applicability and is suitable for oxygen-sensitive test scenarios.

**Table 1:** Physical test parameters and indicators.

Natural moisture content (%)	Dry density (g·cm^−3^)	Void ratio	Plastic limit (%)	Liquid limit (%)	Plasticity index
4.2-11.49	1.31–1.38	1.03	13.78	28.32	14.54

**Fig. 3:**
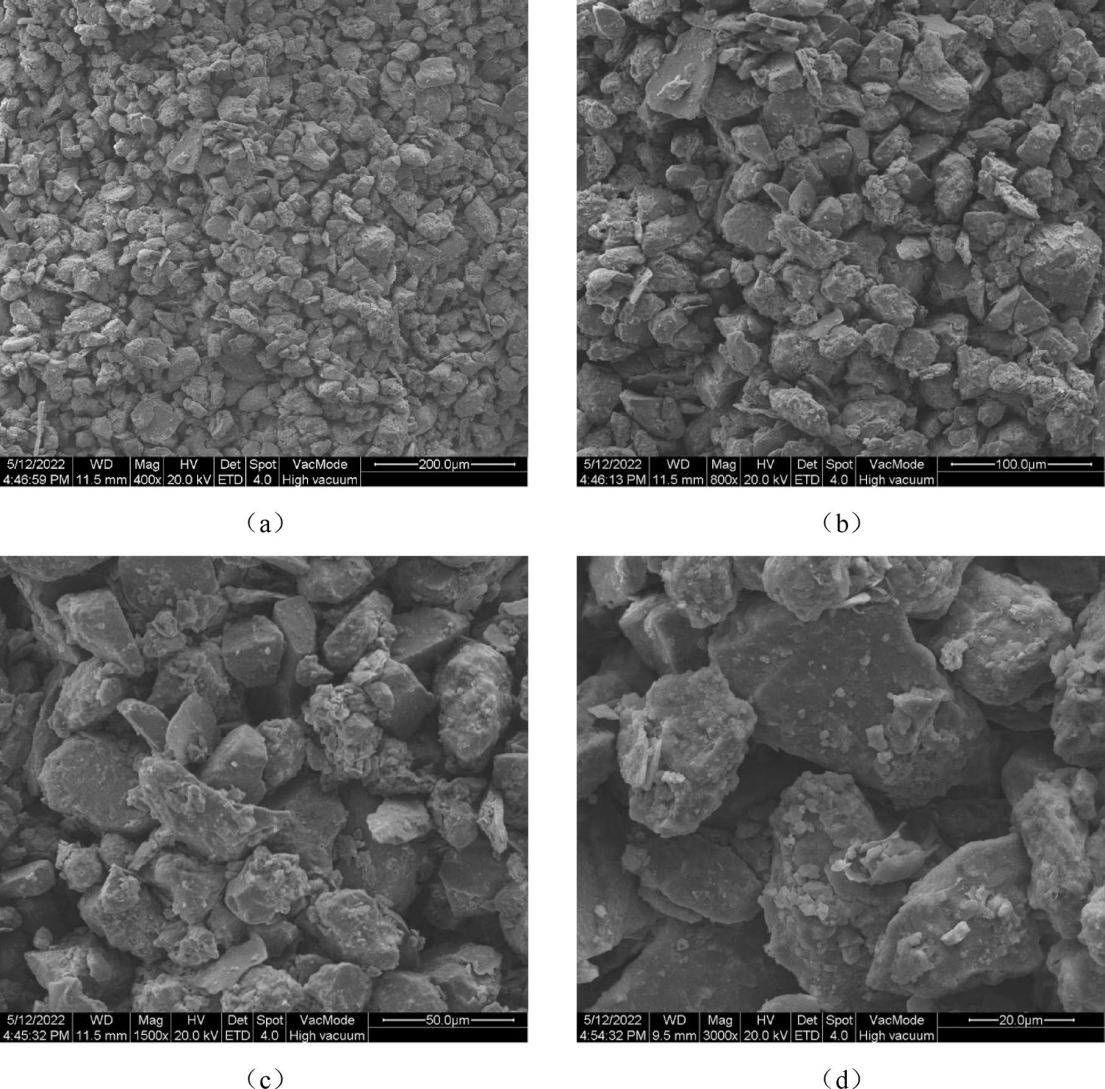
SEM images at different magnifications: (**a**) 400×; (**b**) 800×; (**c**) 1500×; (**d**) 3000×.

A Bettersize2000 laser particle size analyzer (Dandong Baite Instrument Co., Ltd.) was used to analyze the particle size of the sample. Four scanning electron microscopy (SEM) images of the undisturbed sand and loess in Malian City with different magnifications are presented in Fig. 3. There are more angular and semi-angular particles with diameters ≥ 0.05 mm in the sample, and the details of the skeleton particles are clear. There is more surface contact between sand and loess particles, albeit mainly point contacts, which dictate the weak bonding state. The particle arrangement is mainly an overhead structure.

### Instrumentation and methods

**Fig. 4:**
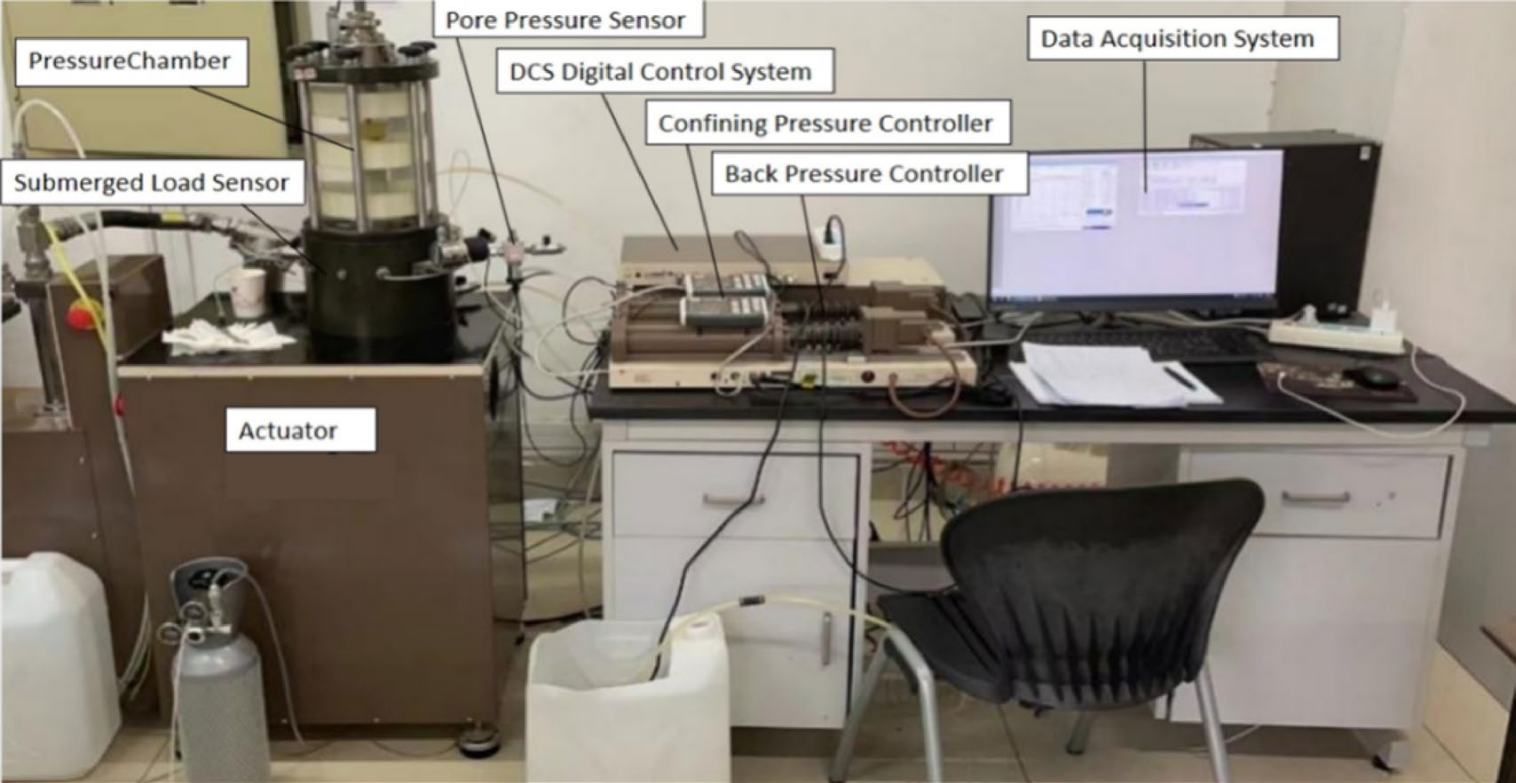
Dynamic triaxial instrument.

**Fig. 5:**
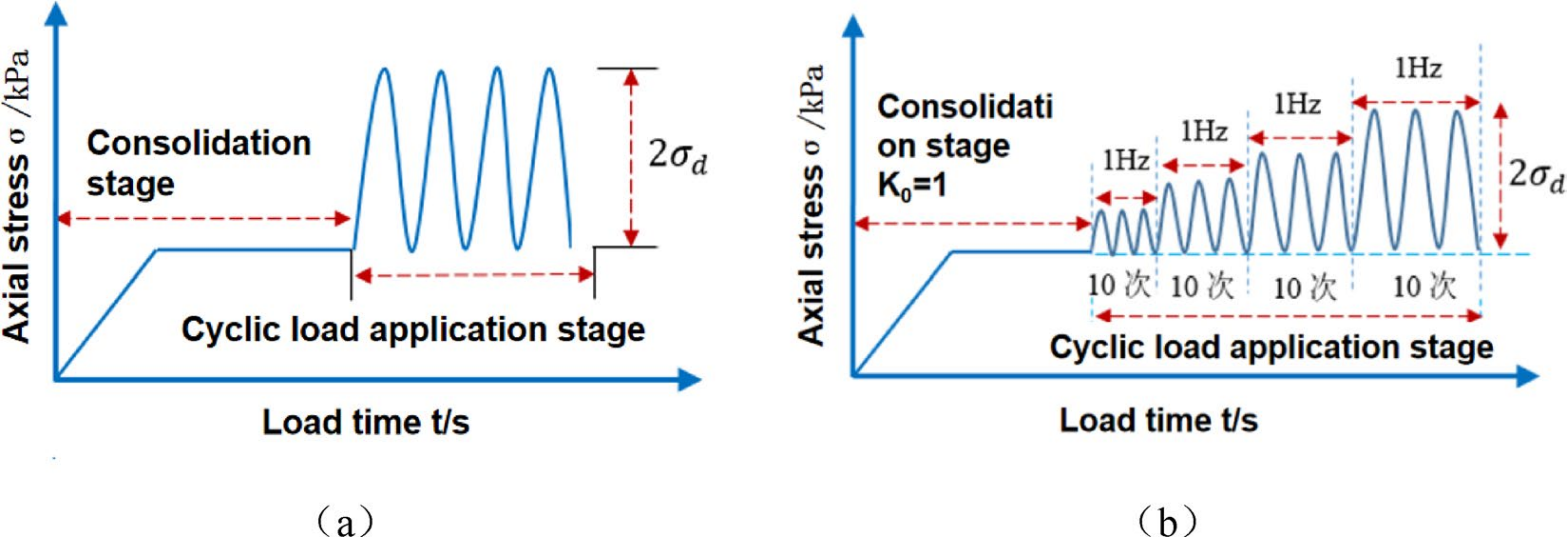
Schematic diagrams of the loading process of dynamic triaxial tests (taking 1 Hz as an example): (**a**) stress control single-stage loading; (**b**) stress control graded loading.

This study used a GDS Dynamic Triaxial Testing System from the UK, as shown in Fig. 4. Its technical specifications included a peak axial dynamic stress of 25 kN, a wide frequency domain control capability of 0.1–5 Hz, and support for programming typical cyclic load waveforms, such as sine waves. The equipment has high accuracy and can reduce disturbance factors during the experimental process. The vibrations caused by an oil drilling rig can be approximated as a sine wave, and therefore, this waveform was selected for the loading experiments. The loading diagram is shown in Fig. 5.

**Fig. 6:**
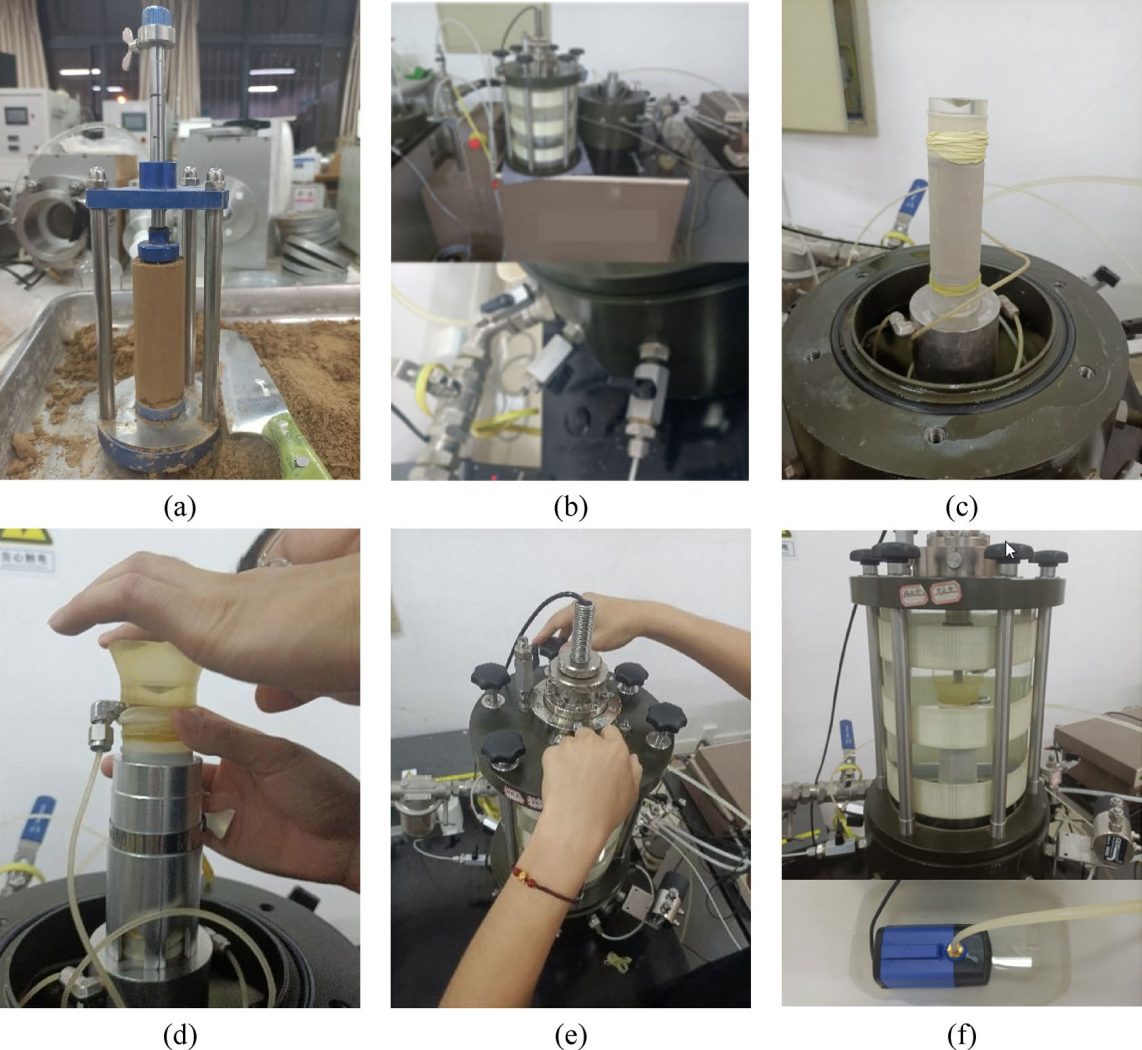
Experimental process: (**a**) soil cutting; (**b**) pipeline and pressure chamber inspection; (**c**) sample installation; (**d**) sample cap installation; (**e**) pressure chamber screw adjustment; (**f**) water injection into the enclosure chamber.

The main experimental process is illustrated in Fig. 6.

The area studied herein is in Yan’an City. A landslide directly related to the vibrations of an oil drilling rig occurred in the well site of Maliancheng Village, Wuqi County, Yan’an City. The low-frequency vibrations generated by the oil drilling rig can easily overlap with the natural frequency (1–10 Hz) of the shallow loose soil layer (e.g., loess). This may cause the vibrational wave to propagate through the stratum, thereby weakening the cohesive force between the soil particles, accelerating crack propagation, and increasing the pore water pressure (if there is groundwater), ultimately resulting in a landslide. Therefore, according to the actual landslide engineering situation and the stratigraphic characteristics of the loess layer in this area, 1, 3, and 5 Hz were selected as the vibrational frequencies tested in the experiments.

In these tests, the undrained consolidation of undisturbed sand loess was carried out using a consolidation method. In the consolidation stage, the sample was in an undrained condition. This means that during the consolidation process, the pore water inside the sample cannot be discharged, and the change in the sample volume is mainly due to the compression of the pore gas when adapting to the increased effective stress. This drainage condition simulates the poor drainage of soil under loading in some practical engineering cases, which is helpful for studying the mechanical behavior and consolidation characteristics of undrained soil.

The experimental process followed two approaches: graded loading and single-stage loading. When applying short-term dynamic loads, graded loading was used. The confining pressure was set to 60 kPa, the number of dynamic cycles *N* was 10, and the cyclic stress amplitude range was 6–60 kPa, meaning that each level of dynamic load increased by 6 kPa. The dynamic stress-strain relationship was computed under cyclic loading to obtain dynamic parameters. The number of vibrations each time was 10 (equivalent to the number of cycles when the predetermined dynamic stress amplitude is reached), and 30 data points were recorded. The moisture content was set to four gradients of 4%, 8%, 12%, and 16%, and the vibrational frequency was set to 1, 3, and 5 Hz. The experimental plan for applying short-term dynamic loads is presented in Table 2. When applying long-term cyclic dynamic loads, single-stage loading was used. The number of dynamic stress cycles *N* was 10,000, the vibrational frequency was 5 Hz, and the cyclic stress amplitudes were 12, 24, 36, 48, and 60 kPa. The experimental plan for applying long-term cyclic dynamic loads is presented in Table 3.

**Table 2:** Short term dynamic load loading scheme.

Confining pressure (kPa)	Moisture content (%)	Frequency (Hz)	Dynamic stress amplitude (kPa)	Number of cycles of vibration per level (*N*)
60	4	1	6, 12, 18, 24, 30, 36, 42, 48, 54, 60	10
		3		
		5		
60	8	1	6, 12, 18, 24, 30, 36, 42, 48, 54, 60	10
		3		
		5		
60	12	1	6, 12, 18, 24, 30, 36, 42, 48, 54, 60	10
		3		
		5		
60	16	1	6, 12, 18, 24, 30, 36, 42, 48, 54, 60	10
		3		
		5		

**Table 3:** Long term dynamic load loading scheme.

Confining pressure (kPa)	Frequency (Hz)	Dynamic stress amplitude (kPa)	Number of cycles of vibration per level (*N*)
60	5	12、24、36、48、60	10,000

## Results

### Dynamic stress-strain relationship

The dynamic stress-strain relationship of loess can reveal the basic relationship characterizing the dynamic mechanical characteristics of loess. Thus, a foundation is laid for analyzing a series of characteristics, including the dynamic shear modulus and damping ratio of loess.

#### Influence of moisture content on dynamic stress and strain

The relationship between the maximum dynamic stress and the dynamic strain can be obtained by connecting the vertices of the stress-strain relationship curve (i.e., hysteresis loop) of the soil under the same consolidation pressure under different dynamic stress amplitudes. The dynamic stress-strain relationship of soil can usually be described by a Hardin-Drnevich hyperbolic model, as expressed in Eq. (1),

1 $${\tau _d}=\frac{{{G_d} \cdot {\gamma _d}}}{{1+\frac{{{\gamma _d}}}{{{\gamma _{\hbox{max} }}}}}}$$

where $\:{\tau\:}_{d}$ is the dynamic shear stress; $\:{G}_{d}$ is the dynamic shear modulus; $\:{\gamma\:}_{d}$ is the dynamic shear stress; and $\:{\gamma\:}_{max}$ is the dynamic shear stress.

**Fig. 7:**
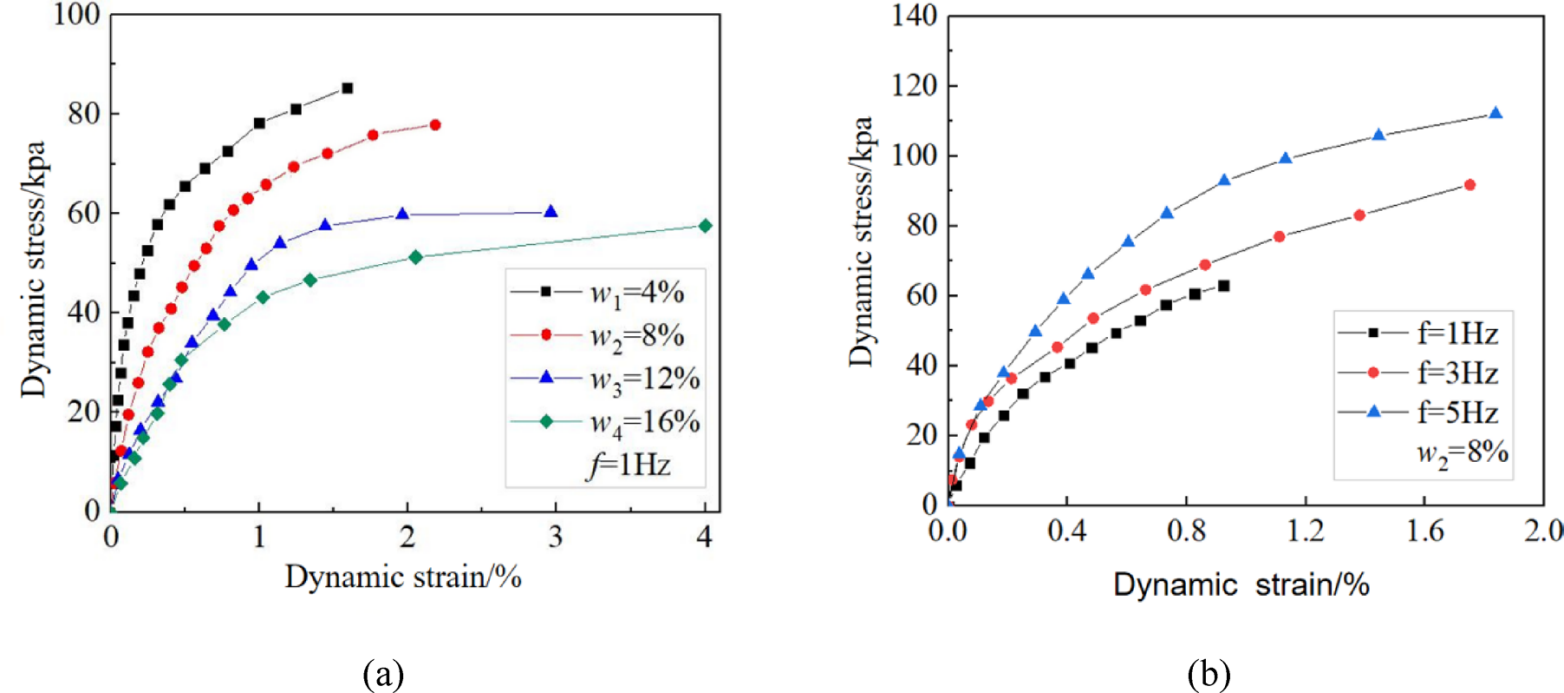
Dynamic stress-strain responses under (**a**) varying moisture contents at 1 Hz and (**b**) varying vibrational frequency conditions at 8% moisture content.

Figure 7a shows the dynamic stress-strain relationship at different moisture contents when the vibrational frequency is 1 Hz. Under the same moisture content conditions, the dynamic stress and strain exhibit a nonlinear growth relationship. Analyzing the geometric characteristics of the stress-strain curve reveals that the tangent modulus is relatively high during the early stage of loading. However, as the number of cyclic loads increases, the internal damage to the specimen gradually accumulates, leading to the continuous development of residual deformation. During this process, the tangent modulus of the curve decays gradually and eventually stabilizes. The evolution of this mechanical response indicates that the initial increment of dynamic strain is small; however, under continuously increasing cyclic load amplitudes, the growth rate of dynamic strain increases significantly until the specimen becomes unstable and fails. Comparing the stress-strain curves of samples with varying moisture contents revealed that under the same cyclic stress amplitude, the stress-strain curve exhibited more significant amplification in samples with higher moisture content. The lubrication effect of the pore water film may weaken the frictional impedance between soil particles, and the penetration of water molecules may weaken the bonding effect between particles. This dual effect significantly reduces the deformation resistance of the soil. Therefore, under the same dynamic stress amplitude, soil with high moisture content will produce a higher strain response. The backbone curves under 12% and 16% moisture contents almost overlap during the initial loading stage, indicating that the dynamic stress-strain relationships of the unsaturated sandy loess samples are less affected by moisture content after the moisture content exceeds a certain threshold.

#### Impacts of vibrational frequency on dynamic stress and strain

Figure 7b shows the dynamic stress–strain relationship at different vibrational frequencies when the moisture content is 8%. Under the same dynamic stress amplitude conditions, there is a significant correlation between vibrational frequency and dynamic strain. Under high-frequency loading, there is limited soil particle displacement due to the time effect limitation of stress wave propagation, and the contact stress between particles has not reached a critical state. In contrast, low-frequency loading conditions provide sufficient time for soil particle reconstruction, which allows cumulative plastic deformation, leading to more significant strain response characteristics. When the vibrational frequency is 3–5 Hz, the dynamic stress-strain curves essentially overlap at the start of the loading process, indicating that the dynamic stress-strain relationship of unsaturated sand loess has a limited influence.

### Dynamic shear modulus

#### Influence of moisture content on dynamic shear modulus

The variation in the dynamic shear modulus of soil as a function of dynamic shear strain can be described by the Hardin-Drnevich hyperbolic model, as expressed in Eq. (2),

2 $${G_d}=\frac{{{G_{\hbox{max} }}}}{{1+\frac{{{\gamma _d}}}{{{\gamma _{ref}}}}}}$$

where $\:{G}_{d}$ is the dynamic shear modulus; $\:{G}_{max}$ is the maximum shear modulus, which usually corresponds to the initial shear modulus under small strain; $\:{\gamma\:}_{d}$ is the dynamic shear strain; and $\:{\gamma\:}_{ref}$ is the reference shear strain.

**Fig. 8:**
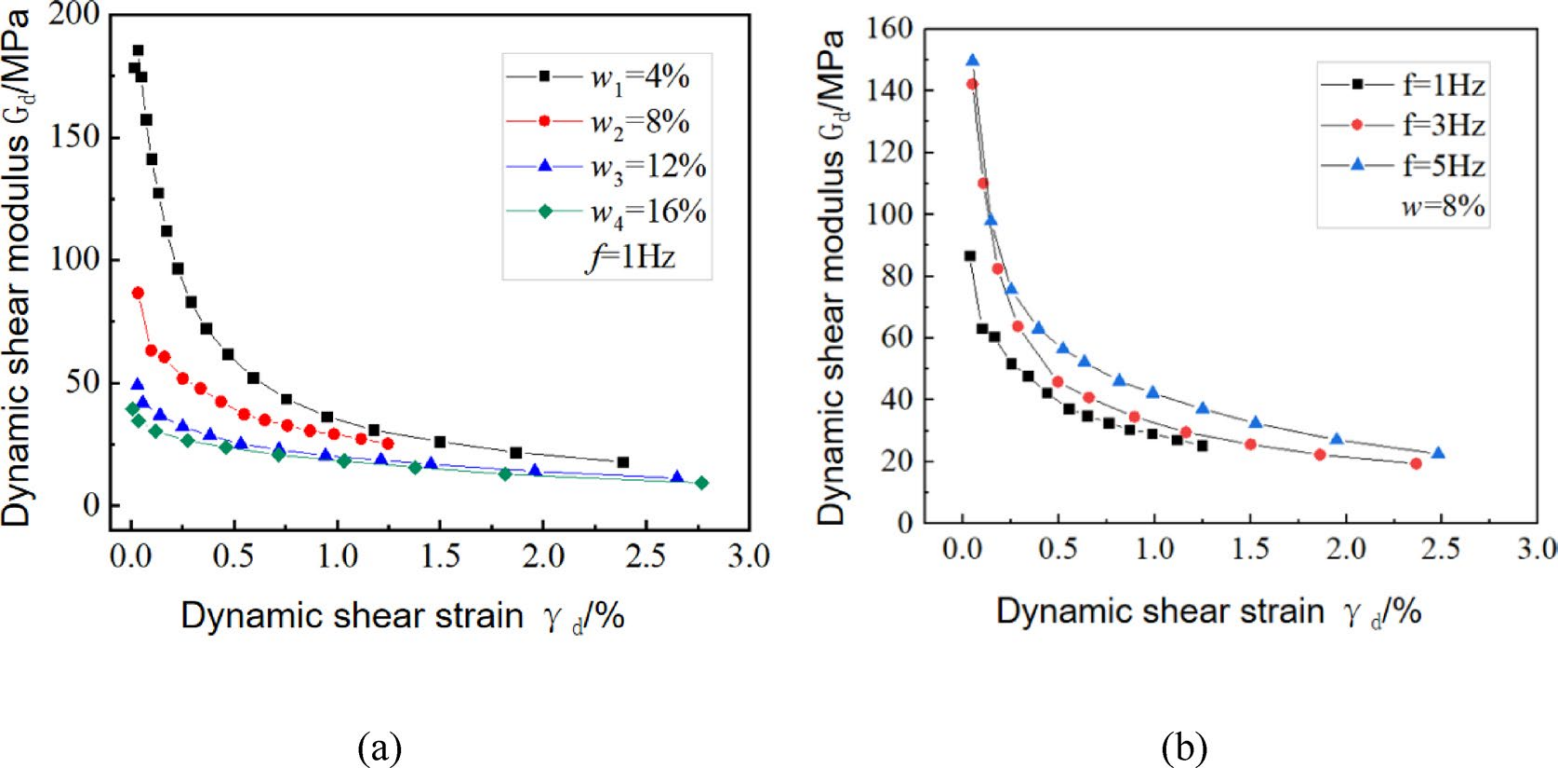
Dynamic shear modulus dependence on moisture content-to-vibrational frequency ratio: (**a**) moisture content-dependent dynamic shear modulus-shear strain interdependence at 1 Hz vibrational frequency; (**b**) G-γ response curves under 8% moisture content across vibrational frequencies.

Figure 8a shows the dynamic mechanical response characteristics of sandy loess under different moisture contents at a vibrational frequency of 1 Hz. Experimental data indicate a significant negative correlation between the amplitude of the shear strain and the dynamic shear modulus of materials. Specifically, as the strain amplitude increases, the modulus gradually decreases according to a logarithmic function. Under cyclic loading, the internal structure of the test specimen evolved from elastic deformation to plastic failure. Furthermore, increasing the moisture content significantly weakened the intergranular bonding characteristics of soil particles. Based on the mechanical response mechanism, the attenuation of the intergranular binding force directly reduces the soil shear resistance capacity, resulting in a consistent reduction of the dynamic shear modulus. Notably, moisture weakens the mechanical parameters and reduces the modulus under cyclic loading, and these coupled effects jointly influence the deformation of soil under dynamic loading. From a microstructural perspective, moisture content beyond the plastic limit mainly exists in large pores, and therefore, its contribution to the weakening of intergranular friction and bonding is limited. Under cyclic loading, moisture content-dependent dynamic shear modulus-shear strain curves exhibit progressive convergence. The dynamic stress-strain curves at 12% and 16% moisture contents are similar, indicating that after the moisture content exceeds a given threshold, the softening effect of water on the soil structure is less impacted by the moisture content. This leads to dynamic shear modulus convergence across specimens during dynamic cyclic loading.

#### Influence of vibrational frequency on dynamic shear modulus

Figure 8b shows the relationship between the dynamic shear strain and the dynamic shear modulus under different vibrational frequencies when the moisture content is 8%. This plot indicates an inverse correlation between vibrational frequency and peak dynamic shear modulus. In general, as the vibrational frequency increases, the time for each vibration is not enough to cause sufficient displacement of soil particles, resulting in slow development of soil strain. The peak dynamic shear moduli of the curves corresponding to 3 and 5 Hz conditions are similar, while the progressive reduction in the dynamic shear modulus-strain interdependence at 1 Hz is gentler than those at 3 and 5 Hz. This is because higher frequencies require greater dynamic shear stress to induce equivalent shear strain. With a larger cyclic stress amplitude, a vibrational cyclic load is applied to the soil in fewer cycles, causing the soil to rapidly progress from elastic deformation to plastic deformation, ultimately leading to a faster decrease in dynamic shear modulus.

### Dynamic damping ratio

#### Influence of moisture content on damping ratio

**Fig. 9:**
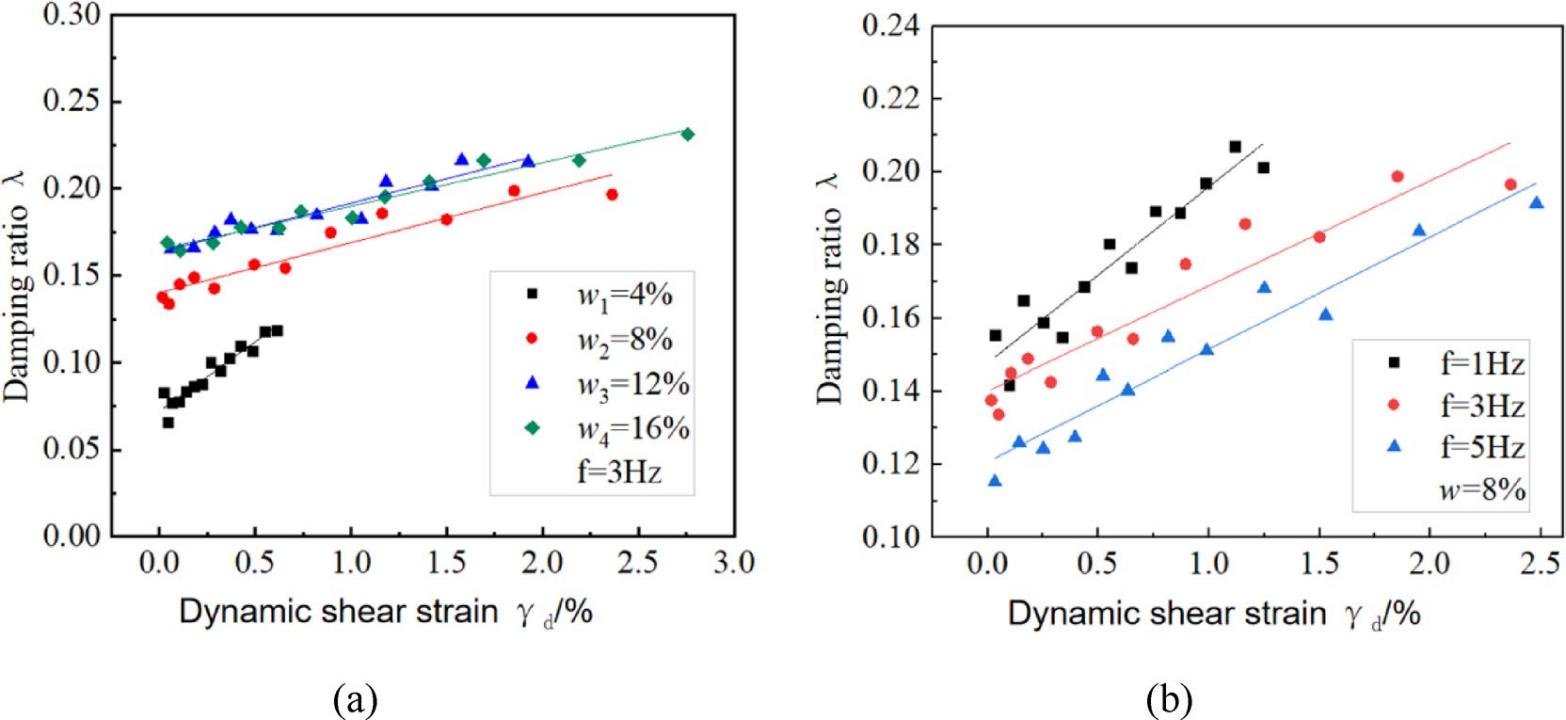
Moisture content-frequency synergistic effects on damping ratio evolution: (**a**) λ-γ response curves at 3 Hz across moisture contents; (**b**) λ-γ response curves at 8% moisture content.

Figure 9a shows the relationship between the dynamic shear strain and the damping ratio under different moisture content conditions at a vibrational frequency of 3 Hz. The damping ratio of undisturbed unsaturated sandy loess generally remains between 0.12 and 0.28, with a few points lower than 0.1. Increasing the dynamic shear strain increases the damping ratio. Comparing the scatter distribution under various moisture contents reveals that the dynamic damping ratio also increases with increasing moisture content. Water has a softening effect on the soil structure, which reduces the cohesion between particles. As a result, particles are more prone to displacement, which increases the energy dissipation of the soil. The slope of the curve under 4% moisture content is larger than that under the other moisture content conditions tested. At lower moisture content, the soil structure is less affected by the limited moisture, and the development of dynamic strain is slow, resulting in a smaller range of dissipated energy. In contrast, when the moisture content is high, the dynamic strain develops more rapidly, and the dissipated energy range is large. When the strain range is small, the dissipated energy is not significant. The curves corresponding to 12% and 16% moisture contents almost overlap, indicating that after the moisture content exceeds the plastic limit, the dynamic damping ratio of the soil exhibits moisture-independent behavior under cyclic loading conditions.

#### Influence of vibrational frequency on damping ratio

Figure 9b shows the relationship between the dynamic shear strain and the damping ratio at different vibrational frequencies when the moisture content is 8%. The damping ratio remains between 0.12 and 2.21, and the vibrational frequency does not exhibit normalization relative to the damping ratio; however, the damping ratio is high at low vibrational frequencies. This suggests that the dynamic stress at low vibrational frequencies is not fully developed, and stress propagation is insufficient when cyclic loads are applied, resulting in low energy dissipation.

## Discussion

The experimental results indicate a threshold effect of the water content at the plastic limit (PL ≈ 14%). Below this threshold, increasing the water content significantly reduces the shear modulus and the damping ratio, consistent with previous reports^14,19^ involving unsaturated soil. Moreover, the water increment above the plastic limit mainly exists in the macropores, meaning that it has a limited contribution to the weakening of intergranular friction and cementation. Therefore, the dynamic parameters tend to converge at 12% and 16% water contents. This phenomenon is verified in sandy loess for the first time in this study. In terms of vibrational frequency, the displacement of soil particles under high-frequency loading is insufficient, consistent with observations using French loess^25^. Moreover, the present study quantified the mechanical response of sandy loess under high-frequency vibrations (1–5 Hz) for the first time and directly simulated the vibrational conditions of an oil drilling rig. The experimental results suggested that there is no clear rule governing the relationship between frequency and damping ratio. The three-stage strain evolution of the cumulative strain is consistent with the model proposed by Gu^21^, thus confirming the universality of loess deformation under cyclic loading. Under a dynamic stress of 60 kPa, the stability requires 3000 cycles; these results can explain the phenomenon of “delayed landslides” in oilfield engineering.

In practical engineering applications, it is crucial to monitor the water content of the site to ensure that the water content of the slope remains ≤ 14% (plastic limit). This enhances the anti-deformation ability and reduces the probability of disaster occurrence. The frequency of drilling rig equipment is controlled and typically suitable for operation under ≥ 3 Hz. It is therefore necessary to increase monitoring under low-frequency vibrations to avoid long-term vibrations. Because they are more likely to cause large deformations, high-vibration projects (e.g., drilling rig foundation) should reserve deformation space according to the dynamic amplitude. According to the three-stage deformation monitoring mechanism, the soil state can be monitored by installing real-time strain sensors to reduce the probability of accidents.

This report describes innovative experiments that fill the knowledge gap regarding the dynamic characteristics of sandy loess. Moreover, the mechanisms and mutual influences governing the water content threshold and frequency are elucidated. The three-stage model of cumulative strain proposed herein provides direct theoretical support for engineering disaster prevention in loess areas. This core contribution can translate laboratory results into practical engineering parameters, such as slope maintenance and vibrational control. These actionable insights can be applied immediately to promote the prevention and control of loess disasters in oil fields, transportation, and other fields. However, the impacts of the confining pressure and consolidation stress ratio on the dynamic stress strain and modulus have not been discussed. Future studies should therefore supplement multi-stress path tests, and the sand loess in other regions of Northwest China (e.g., Longdong) should be verified.

## Conclusions

This study investigated undisturbed unsaturated sandy loess near the back wall of the Maliancheng Village landslide in Yan’an City. A GDS Dynamic Triaxial Testing System was used to monitor the dynamic stress-strain relationships, dynamic shear moduli, damping ratios, and cumulative strains under high-frequency cyclic loading. The results are as follows:

1. Upon reaching the plastic limit of the soil sample in terms of moisture content, the dynamic shear modulus, dynamic stress-strain relationship, and dynamic damping ratio of unsaturated sandy loess show little-to-no influence from additional moisture content.
2. Before reaching the plastic limit in terms of moisture content, the moisture content has a significant impact on the dynamic shear modulus, dynamic stress-strain relationship, and dynamic damping ratio of unsaturated sandy loess. As the moisture content increases, the dynamic stress-strain relationship changes more rapidly, and under the same shear strain conditions, the dynamic shear modulus decreases steadily, while the dynamic damping ratio increases gradually.
3. As the vibrational frequency increases, the dynamic strain of unsaturated sandy loess decreases, while the dynamic stress varies; both the overall trend and the maximum dynamic shear modulus progressively decrease. The dynamic damping ratio does not exhibit normalization, but rather, the overall trend decreases. The impacts of high vibrational frequencies on the dynamic shear modulus, dynamic stress-strain relationship, and dynamic damping ratio of unsaturated sandy loess are relatively small during the initial loading stage.
4. The cumulative strain of unsaturated sandy loess under high vibrational frequency undergoes exponential growth, gradual evolution, and dynamic equilibrium, corresponding to soil subsidence characteristics. As the cyclic stress amplitude increases, the stability frequency of unsaturated sandy loess gradually increases. When the cyclic stress amplitude increases to 60 kPa, the vibrational frequency stabilize after approximately 3000 cycles.

## Data Availability

Data is provided within the manuscript： The data that support the findings of this research are available at https://www.cnki.net.
